# Tumor-infiltrating lymphocytes are the key determinants of pathological features associated with pathogenic *BRCA* variants in high-grade serous ovarian carcinoma

**DOI:** 10.3389/fmed.2025.1555883

**Published:** 2025-06-03

**Authors:** Dang H. Nguyen-Phan, Tin Dang, Anh N. Dang, Loc T. H. Huynh, Phuong T. B. Nguyen, Vu Q. Tran, Hanh T. T. Ngo, Thao T. P. Doan, Tu A. Thai, Chien-Chin Chen

**Affiliations:** ^1^Department of Pathology and Forensic Medicine, Pham Ngoc Thach University of Medicine, Ho Chi Minh City, Vietnam; ^2^Department of Pathology, Ho Chi Minh City Oncology Hospital, Ho Chi Minh City, Vietnam; ^3^University of Medicine and Pharmacy at Ho Chi Minh City, Ho Chi Minh City, Vietnam; ^4^Department of Pathology, Ho Chi Minh City University Medical Center, Ho Chi Minh City, Vietnam; ^5^Department of Pathology, Ditmanson Medical Foundation Chia-Yi Christian Hospital, Chiayi, Taiwan; ^6^Department of Cosmetic Science, Chia Nan University of Pharmacy and Science, Tainan, Taiwan; ^7^Doctoral Program in Translational Medicine, National Chung Hsing University, Taichung, Taiwan; ^8^Department of Biotechnology and Bioindustry Sciences, College of Bioscience and Biotechnology, National Cheng Kung University, Tainan, Taiwan

**Keywords:** BRCA, histology, high-grade serous ovarian carcinoma, mitosis, ovary, tumor infiltrating lymphocyte

## Abstract

**Background:**

High-grade serous ovarian carcinoma (HGSOC), an aggressive cancer associated with pathogenic *BRCA* variants, causes genomic instability and sensitivity to poly (ADP-ribose) polymerase inhibitors. Identifying pathogenic *BRCA* variants is crucial for the treatment of HGSOC; however, genetic testing is expensive and time-consuming. This study aimed to explore pathological features, particularly the presence of tumor-infiltrating lymphocytes (TILs), as potential surrogates to streamline patient selection for genetic testing.

**Methods:**

We retrospectively analyzed 58 cases of HGSOC with known *BRCA* variant profiles. Tumors were categorized as TIL-positive or TIL-negative based on the presence of > 40 or ≤ 40 intraepithelial lymphocytes in a single high-power field (HPF), respectively. Key pathological features, including solid, endometrioid, and transitional (SET) architecture patterns; necrosis; and mitotic activity, were evaluated within these subgroups. Statistical analyses were used to determine the associations between these features and *BRCA* variant status.

**Results:**

In TIL-negative HGSOCs, SET patterns were strongly associated with pathogenic or likely pathogenic *BRCA* variants (*p* = 0.028), emerging as the most reliable morphological marker in this group. In TIL-positive HGSOCs, low mitotic activity (≤7 mitotic figure per 10 HPFs) was significantly correlated with pathogenic *BRCA* variants (*p* = 0.0002), underscoring its diagnostic significance. Necrosis and mitotic activity in TIL-negative cases and SET patterns in TIL-positive cases were not significantly associated with pathogenic *BRCA* variants. Combined analysis of both TIL subgroups diluted these associations, underscoring the significance of stratifying cases by the immune context.

**Discussion:**

The presence of TILs affects the diagnostic value of pathological features for *BRCA* variant status in HGSOC. Regarding pathogenic *BRCA* variants, SET patterns and low mitotic activity were identified as critical markers in TIL-negative tumors and TIL-positive tumors, respectively. These associations likely stem from interactions among genomic instability, immune response, and tumor growth. Our framework leverages these insights to prioritize high-risk cases for genetic testing, thereby optimizing resource allocation.

**Conclusion:**

The presence of TILs is critical for understanding the association between pathological features and pathogenic *BRCA* variants in HGSOC. To improve pathogenic *BRCA* variant prediction, optimize genetic testing, and guide tailored intervention, our framework integrates immune context and morphological markers. This approach is especially useful in resource-limited settings and can enhance diagnostic efficiency and clinical decision-making.

## 1 Introduction

Ovarian cancer is the third most prevalent cancer among females of childbearing age (1). Among its subtypes, the most common and deadliest form is high-grade serous ovarian carcinoma (HGSOC), which is responsible for most ovarian cancer-related deaths (2, 3). The aggressive nature of HGSOC, along with its frequent late-stage diagnosis, highlights the significance of refined diagnostic and prognostic tools for guiding therapeutic interventions (4).

*BRCA1* and *BRCA2* play a significant role in the homologous recombination repair of DNA double-strand breaks (5). Approximately 20–25% of patients with HGSOC exhibit germline or somatic mutations in *BRCA1* and *BRCA2* (6, 7). The pathogenic variants of these genes cause homologous recombination deficiency (HRD), subsequently leading to genetic instability, increased mutation burden, and sensitivity to DNA-damaging agents, including platinum-based chemotherapy and poly (ADP-ribose) polymerase (PARP) inhibitors (8, 9). PARP inhibitors have been reported to significantly improve progression-free survival in patients with HGSOC with pathogenic *BRCA* variants (10–12). Current guidelines recommend *BRCA1/2* and HRD testing at the time of primary diagnosis, ideally before completing first-line chemotherapy, because up to 50% of patients with ovarian cancer demonstrate HRD (13). Early detection is crucial for precision medicine. Despite its essential clinical implication, identifying *BRCA* variant status can be challenging in resource-limited settings, where next-generation sequencing (NGS) may be costly and inaccessible to several patients (14, 15). Although females with ovarian cancer are recommended to undergo testing (16, 17), stratifying patients into risk categories for pathogenic *BRCA* variants is an alternative approach (18, 19). Individuals with a higher risk profile should be prioritized for genetic testing. Morphological features offer a cost-effective way to identify patients at high risk for pathogenic *BRCA* variants; this approach has been well established in breast cancer research (20–23). Such approaches prioritize genetic testing for patients with a high likelihood of benefiting from the test, enabling better allocation of resources (19).

The tumor microenvironment profoundly impacts tumor growth and treatment response (24). Tumor-infiltrating lymphocytes (TILs) are key factors in the tumor microenvironment. The presence of TILs reflects the immune system’s ability to recognize and respond to tumor cells. TILs are frequently associated with better outcomes in cancer, including HGSOC (25, 26), reflecting more robust immune surveillance and stronger antitumor response. Simultaneously, *BRCA1/2*-regulated autophagy affects immune response by modulating MHC class II expression and hindering immune recognition of clonal neoantigens (27). Considering the cellular immunity TILs predict prognosis and may indicate underlying *BRCA1/2* alterations that facilitate immune evasion. Furthermore, we hypothesize that TILs can affect the expression of specific morphological features. Stratifying HGSOCs by the presence of TILs may offer an opportunity to more systematically explore these relationships and refine their use in predicting *BRCA* variant status.

Several studies have been conducted to identify pathogenic *BRCA* variant-related pathological features in ovarian cancer (18, 28–30). These studies have identified solid, endometrioid, and transitional architecture (SET) patterns; mitotic activity; and necrosis as key morphological features in pathogenic *BRCA* variant-related HGSOC. However, the significance of these pathological features varies across studies, and their predictive value in the context of TIL stratification remains underexplored. Therefore, the present study investigated the relationship between *BRCA* variant status and critical morphological features (SET patterns, necrosis, and mitotic activity) across TIL-positive and TIL-negative HGSOC tumors. We also determined whether stratification according to the presence of TILs improved the diagnostic utility of these features, thereby refining patient selection for genetic testing and enhancing clinical decision-making in settings where comprehensive *BRCA* sequencing may not be readily available.

## 2 Materials and methods

### 2.1 Ethical approval and patient recruitment

The Ho Chi Minh City Medicine and Pharmacy University Ethics Committee approved this study (approval number: IRB-VN01002). All procedures were conducted following institutional and international ethical standards. Data of patients with HGSOC were retrospectively collected from Ho Chi Minh City Oncology Hospital over 20 months (August 2022–April 2024), with prior targeted NGS testing for *BRCA1/2* variant profiles. This study used archived formalin-fixed paraffin-embedded (FFPE) tissue blocks, which were fully anonymized before analysis to ensure patient confidentiality. Patients with HGSOC were included in this study when their samples (tissue FFPE blocks and slides) were available and of good quality to undergo morphological feature analysis. Additional clinical data, including age at diagnosis and disease stage, were collected for each case.

### 2.2 Sequencing for BRCA1/2 variants

Regarding targeted NGS testing for *BRCA1/2*, all recruited patients with HGSOC were tested at the Ho Chi Minh City Oncology Hospital. In brief, genomic DNA was extracted from tumor-bearing FFPE tissues using the GeneRead™ DNA FFPE Kit (Qiagen, Hilden, Germany). NGS was performed on the Illumina MiSeqDx platform using the reagent kit BRCAaccuTest™ Plus (NGeneBio Co., Ltd., Seoul, South Korea) to produce libraries targeting *BRCA1* and *BRCA2*. Sequencing data were processed and analyzed using the automated NGeneAnalySys™ Software (NGeneBio Co., Ltd., Seoul, South Korea), with variants classified according to the American College of Medical Genetics and Genomics guidelines into the following categories: pathogenic, likely pathogenic, uncertain significance, likely benign, and benign variants (31). In the present study, cases with pathogenic or likely pathogenic variants were classified into the pathogenic group, whereas cases with benign or likely benign variants were categorized into the benign group. Variants of uncertain significance were excluded from the analysis.

### 2.3 Assessment of pathological features and TILs in the tumor microenvironment

We retrospectively retrieved tumor-containing slides from each case to assess pathological characteristics. For cases where existing slides were faded or unsuitable for assessment, new hematoxylin and eosin (H&E)-stained slides were subsequently prepared from the corresponding tissue blocks to ensure diagnostic quality. We collected 184 slides of tissue samples from all 58 cases of HGSOC analyzed by experienced pathologists to independently evaluate pathological features. The number of slides per case ranged from 1 to 9, with the mean number of slides per case being 3.17. Furthermore, we employed validation methods to minimize interobserver bias for morphological assessments. Before the evaluation, we reached a consensus on the morphology assessment as this study is an integrative teamwork. Each case was independently reviewed by at least two pathologists. In the event of a disagreement, our central review board would hold a consensus meeting to discuss the findings and reach a joint decision.

**FIGURE 1:**
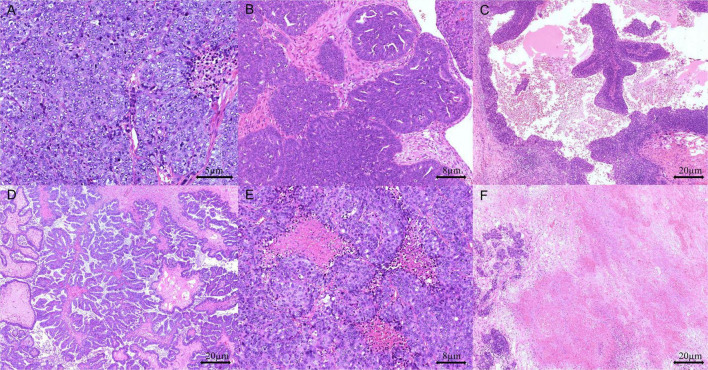
Representative pathological features in high-grade serous ovarian carcinoma (HGSOC): **(A)** solid growth pattern (H&E stain, 200 × magnification), **(B)** endometrioid growth pattern (H&E stain, 100 × magnification), **(C)** transitional cell-like growth pattern (H&E stain, 40 × magnification), **(D)** papillary growth pattern (H&E stain, 40 × magnification), **(E)** comedo-like necrosis (H&E stain, 100 × magnification), and **(F)** geographic necrosis (H&E stain, 40 × magnification).

To assess architectural patterns and other key morphological features, including TILs and comedo-like or geographic necrosis, H&E slides were analyzed under low magnification (40×). Architectural patterns were categorized as solid, endometrioid, transitional cell carcinoma-like, papillary, or micropapillary (Figure 1). Tumors were classified as SET-positive when ≥ 30% of tumor cells displayed solid, endometrioid, or transitional cell carcinoma-like patterns.

**FIGURE 2:**
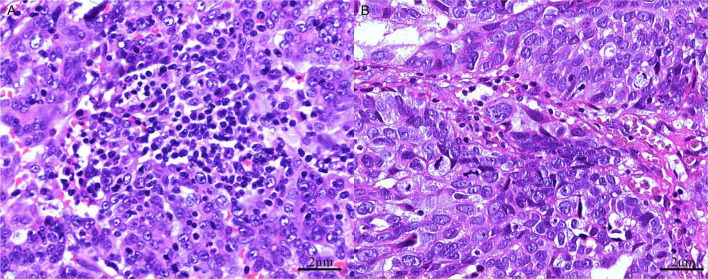
Tumor-infiltrating lymphocytes (TILs) in HGSOC. **(A)** TIL-positive: > 40 dense intraepithelial infiltrates of TILs (H&E stain, 400 × magnification); **(B)** TIL-negative: ≤ 40 sparse infiltrates of TILs (H&E stain, 400 × magnification).

Areas with the highest concentration of TILs or mitotic figures, termed “hot spots,” were identified for evaluation. Mononuclear cells were considered TILs when located within the tumor margins or in the intercellular spaces and the cores of papillary tumor structures. Cells outside the tumor border, those associated with necrotic regions, and those within blood vessels were excluded. TILs were considered positive when the tumor contained more than 40 intraepithelial lymphocytes in at least one high-power-field (HPF) (Figure 2). Furthermore, we classified mitotic activity using the Nottingham Grading System (32), which was originally developed for breast cancer histopathological grading. Using our microscope (Olympus BX43) with a 0.5-mm field diameter, we counted the number of mitotic figures in 10 consecutive HPFs within the most mitotically active area of the tumors and subsequently categorized tumors as low (≤ 7 mitotic figures/10 HPFs), moderate (8–14 mitotic figures/10 HPFs), or high (≥ 15 mitotic figures/10 HPFs) (33).

Despite several studies focusing on TILs and mitotic rates in ovarian cancer, universally accepted evaluation criteria are not available. The methodologies applied here were adapted from established frameworks to ensure consistency and reliability in assessing these critical features (29, 30).

### 2.4 Statistical analysis

Statistical analysis was performed using Statistical Package for the Social Sciences (version 25, IBM, Armonk, NY, United States) and GraphPad Prism 8 software to compare morphological and pathological features between the pathogenic and benign *BRCA* variant groups. Chi-square test was used for evaluating qualitative variables, whereas *t*-test was used for evaluating quantitative variables. When the expected value in any contingency table cell was < 5, Fisher’s exact test was used instead of the chi-square test. For quantitative variables, when normality assumptions were violated (assessed using the Shapiro–Wilk test), the Mann–Whitney U test was applied as a non-parametric alternative. Statistical significance was set at *p* < 0.05 (two-tailed).

## 3 Results

### 3.1 Patients’ age at diagnosis and Federation Internationale de Gynecologie et d’Obstetrique (FIGO) stage

Overall, 58 patients with HGSOC were retrospectively enrolled, and the age at diagnosis was 33–78 years, with a mean age of 55.8 years (standard deviation = 9.1) and a median age of 55.0 years. Patients with benign *BRCA* variants had a mean age of 55.0 years (standard deviation = 9.3) and a median age of 51.0 years, whereas those with pathogenic *BRCA* variants had a mean age of 57.0 years (standard deviation = 8.8) and a median age of 58.0 years. Patients with pathogenic and benign *BRCA* variants showed no statistically significant differences in terms of age (*p* = 0.163; Mann–Whitney U test) (Supplementary Figure 1).

Regarding the FIGO stage, most cases were diagnosed with FIGO IIIC, comprising 72.4% (42/58) of the cohort. Among the patients with benign *BRCA* variants, 24 cases were assigned to FIGO IIIC, whereas 18 cases with pathogenic *BRCA* variants were assigned to this stage. Early stage disease (FIGO IC or IIB) was observed only in cases with benign *BRCA* variants. In contrast, no cases with pathogenic *BRCA* variants were diagnosed at a stage earlier than stage IIIC. Both groups encompassed cases diagnosed at an advanced stage (FIGO IVB).

### 3.2 BRCA1/2 variant profiles and TILs

**FIGURE 3:**
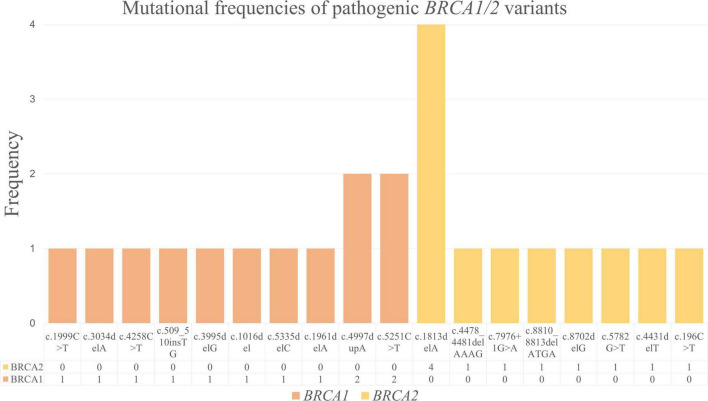
Mutational frequencies of pathogenic *BRCA1/2* variants.

We detected 10 distinct *BRCA1* and 8 distinct *BRCA2* pathogenic variants (Figure 3). Among the pathogenic *BRCA1* variants, c.4997dupA and c.5251C > T were the most frequent; each was observed in 2 of 23 cases. The remaining pathogenic *BRCA1* variants, including c.1999C > T, c.3034delA, c.4258C > T, c.509_510insTG, c.3995delG, c.1016del, c.5335delC, and c.1961delA, were noted in 1 of 23 cases each. Considering the pathogenic *BRCA2* variants, we detected c.1813delA in 4 of 23 cases, making it the most frequent pathogenic variant in our cohort. The other pathogenic *BRCA2* variants, including c.4478_4481delAAAG, c.7976 + 1G > A, c.8810_8813delATGA, c.8702delG, c.5782G > T, c.4431delT, and c.196C > T, were detected in 1 of 23 cases each.

Among the 58 cases of HGSOC analyzed, 30 and 28 were classified as TIL-negative and TIL-positive, respectively. The information and analyses of demographic and clinical characteristics between the two groups are shown in Supplementary Table 1. In the TIL-negative group, 16 cases (53.3%) had benign *BRCA* variants, whereas 14 (46.7%) harbored pathogenic *BRCA* variants. Similarly, in the TIL-positive group, 19 cases (67.9%) had benign *BRCA* variants, whereas 9 (32.1%) had pathogenic *BRCA* variants. No statistical significance in pathogenic *BRCA* variant distribution was observed between the two groups (*p* = 0.29).

### 3.3 Pathological features in the TIL-negative group

**FIGURE 4:**
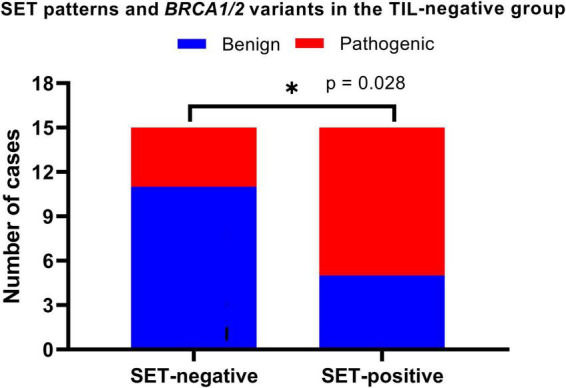
Solid, endometrioid, and transitional (SET) patterns and *BRCA* variants in TIL-negative HGSOCs. SET-positive was defined if ≥ 30% of the tumor components demonstrated solid, endometrioid, or transitional cell carcinoma-like patterns. We found SET-positive tumors positively associated with pathogenic *BRCA* variants (*p* = 0.028). *p < 0.05.

In the TIL-negative group (30 cases of HGSOC), we evaluated the relationship between pathogenic *BRCA* variants and three key morphological features: SET patterns, necrosis, and mitotic activity. Without dominant infiltrates of intraepithelial TILs, we noted a statistically positive association between SET-positive tumors and pathogenic *BRCA* variants. This association was evaluated using the chi-square test, which yielded a relative risk of 2.200 and a *p*-value of 0.028 (Figure 4). Our findings suggest that SET-positive tumors are particularly significant in TIL-negative HGSOCs, where SET patterns are a key morphological marker for identifying pathogenic *BRCA* variants.

Combining comedo and geographic necrosis, we observed necrosis in 19 of the 30 cases (63.3%). However, statistical analysis of necrosis patterns revealed no significant association with *BRCA* variants (*p* = 0.389). Regarding mitotic activity, high mitotic activity was predominant in 10 cases (71.4%) with pathogenic *BRCA* variants, whereas 3 (21.4%) and 1 (7.1%) cases were in the moderate and low categories, respectively. Similarly, among the 16 benign *BRCA* cases, 11 (68.8%), 2 (12.5%), and 3 (18.8%) demonstrated high, low, and moderate activities, respectively. Fisher’s exact test for mitotic activity categories and *BRCA* variants revealed no significant association (*p* = 0.733). Our results indicate that mitotic activity and necrosis do not aid in distinguishing between benign and pathogenic *BRCA* variants in TIL-negative HGSOCs.

### 3.4 Pathological features in the TIL-positive group

The TIL-positive group comprised 28 cases of HGSOC, and we evaluated the relationships between *BRCA* variants and three pathological features (SET patterns, necrosis, and mitotic activity) as we did in the TIL-negative group. In the TIL-positive group, 15 of 28 cases (53.6%) met the criteria for SET-positive tumors. Among the nine cases with pathogenic *BRCA* variants, five were SET-positive cases (55.6%). Similarly, 10 of 19 cases (52.6%) with benign *BRCA* variants were SET-positive. No significant difference in *BRCA* variants was noted between the SET-positive and SET-negative groups, as revealed using Fisher’s exact test (*p* = 1). Our results suggest that SET patterns are unreliable for differentiating *BRCA* variants within the TIL-positive group.

Necrosis was detected in 14 of the 28 cases (50.0%). Among the 9 cases with pathogenic *BRCA* variants, 4 (44.4%) exhibited necrosis, whereas 10 (52.6%) had necrosis in 19 cases with benign *BRCA* variants. Fisher’s exact test was used to evaluate the relationship between necrosis and *BRCA* variant status in TIL-positive tumors. The test yielded a *p*-value of 1, indicating that necrosis and *BRCA* variants were not significantly associated. These findings emphasize that although frequently observed, necrosis patterns do not reliably differentiate between benign and pathogenic *BRCA* variants in TIL-positive tumors.

**FIGURE 5:**
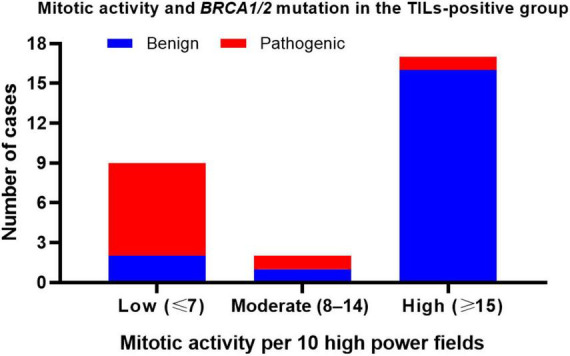
Mitotic activity and *BRCA* variants in TIL-positive HGSOCs. Low mitotic activity is strongly associated with pathogenic *BRCA* variants in TIL-positive HGSOCs.

Interestingly, in TIL-positive tumors, mitotic activity was significantly associated with *BRCA* variant status (Figure 5). Among the nine cases with pathogenic *BRCA* variants, seven (77.8%) exhibited low mitotic activity, one (11.1%) showed high mitotic activity, and one (11.1%) showed moderate mitotic activity. Conversely, in the 19 cases with benign *BRCA* variants, 2 cases (10.5%) exhibited low mitotic activity, 1 case (5.3%) showed moderate mitotic activity, and 16 cases (84.2%) had high mitotic activity.

Classifying tumors with moderate or high mitotic activity as the same group and comparing them to tumors with low mitotic activity using Fisher’s exact test revealed that low mitotic activity was significantly and positively associated with pathogenic *BRCA* variants in TIL-positive tumors (*p* = 0.0002). The relative risk was 4.722. Our results suggest that low mitotic activity is a distinguishing feature of pathogenic *BRCA* variants in TIL-positive tumors.

In summary, among TIL-positive tumors, the most significant morphological predictor of *BRCA* variant status was mitotic activity. Low mitotic activity was strongly associated with pathogenic *BRCA* variants, whereas moderate-to-high mitotic activities were more prevalent in benign cases. In contrast, neither SET patterns nor necrosis was significantly associated with *BRCA* variant status.

### 3.5 Pathological features in the complete dataset

We also assessed the relationships between *BRCA* variant status and key pathological features (SET patterns, necrosis, and mitotic activity) in the complete dataset, including TIL-positive and TIL-negative HGSOCs. Our findings showed a higher frequency of SET positivity in cases with pathogenic *BRCA* variants than in those with benign variants. However, no statistically significant difference was observed (*p* = 0.096). Our findings suggested that although SET patterns exhibit some diagnostic value, their predictive value appears less pronounced when analyzing TIL-positive and TIL-negative tumors together.

Similarly, in the complete dataset, necrosis showed no significant association with *BRCA* variant status (*p* = 0.620), suggesting that necrosis patterns cannot differentiate between benign and pathogenic *BRCA* variants across a mixed population of TIL-positive and TIL-negative tumors. In addition, mitotic activity did not significantly affect *BRCA* variant status in the complete dataset (*p* = 0.075), indicating that the diagnostic value of mitotic activity observed in specific TIL subgroups is not maintained when combined.

The lack of significant associations in the complete dataset highlights the significance of stratifying tumors by dense intraepithelial infiltrates of TILs when evaluating pathological features. The relationships observed in the separate analyses of TIL-positive and TIL-negative groups, including the strong association between SET patterns and pathogenic *BRCA* variants in TIL-negative cases and that between low mitotic activity and pathogenic *BRCA* variants in TIL-positive cases, are obscured when these groups are combined. These data suggest that the diagnostic value of morphological features is context-dependent, and stratifying tumors by immune infiltration is the first step before using other pathological factors as predictive features of *BRCA* variant status. The histopathological features of the cases included in our study in correlation with the *BRCA* variant status are summarized in Table 1.

**TABLE 1:** Correlation of histopathological features with *BRCA1/2* variant status in high-grade serous ovarian carcinoma (HGSOC)

		Pathogenic *BRCA1/2* variant	Benign *BRCA1/2* variant	*p*-value
**TIL-negative**				
SET patterns	Positive	10	5	**0.028**
	Negative	4	11	
Necrosis	Present	10	9	0.389
	Absent	4	7	
Mitotic activity	High	10	11	0.733^†^
	Moderate	3	2	
	Low	1	3	
**TIL-positive**				
SET patterns	Positive	5	10	1
	Negative	4	9	
Necrosis	Present	4	10	1
	Absent	5	9	
Mitotic activity	High	1	16	**0.0002** ^†^
	Moderate	1	1	
	Low	7	2	
**Complete dataset**				
SET patterns	Positive	15	15	0.096
	Negative	8	20	
Necrosis	Present	14	19	0.620
	Absent	9	16	
Mitotic activity	High	11	27	0.075^†^
	Moderate	4	3	
	Low	8	5	

^†^We classified tumors with moderate or high mitotic activity as the same group and compared them to tumors with low mitotic activity using Fisher’s exact test.

## 4 Discussion

### 4.1 General demographic characteristics

In this study, the age range at the time of HGSOC diagnosis was 33–78 years, with a mean of 55.8 years. The mean age aligned with global data (34, 35), suggesting that our cohort is representative of individuals affected by HGSOC. In addition, the FIGO stage distribution supports the representativeness of this cohort. Notably, 72.4% of our cases were diagnosed as stage IIIC, reflecting the aggressive nature of the disease, which is frequently identified at advanced stages owing to its asymptomatic early progression (35). Herein, the typical age distribution and FIGO stage pattern enhance the applicability of our findings to other populations with HGSOC.

### 4.2 TILs in the tumor microenvironment are a key indicator of pathological features

This study investigated the pathological features of HGSOCs concerning *BRCA* variant status, focusing on stratification by the presence of dense intraepithelial TILs. By examining pathological features, including SET patterns, necrosis, and mitotic activity, within the TIL-positive and TIL-negative groups and in the complete dataset, we aimed to identify morphological markers that could differentiate pathogenic *BRCA* variants from benign variants.

In the TIL-negative group, SET patterns were the most reliable predictor for pathogenic *BRCA* variants. A strong association between SET-positive tumors and pathogenic *BRCA* variants was observed (*p* = 0.028), consistent with previous studies that reported associations between SET patterns and *BRCA*-mutated tumors (29, 30, 36, 37). In contrast, necrosis and mitotic activity were not significantly correlated with *BRCA* variant status in this group. These results suggest that SET patterns should be prioritized for evaluation in TIL-negative tumors.

In the TIL-positive group, low mitotic activity was strongly associated with pathogenic *BRCA* variants. Conversely, SET patterns and necrosis were not significantly correlated with *BRCA* variant status. Our results emphasize the significance of mitotic activity as a crucial factor for distinguishing tumors with immune infiltration. Moreover, we analyzed the relationship between the evaluated pathological features (SET patterns, necrosis, and mitotic activity) and pathogenic *BRCA* variants in the entire cohort. No statistically significant associations were observed in the complete dataset, underscoring the significance of stratifying cases by TILs.

### 4.3 Proposed framework for predicting BRCA variant status in individual cases

**FIGURE 6:**
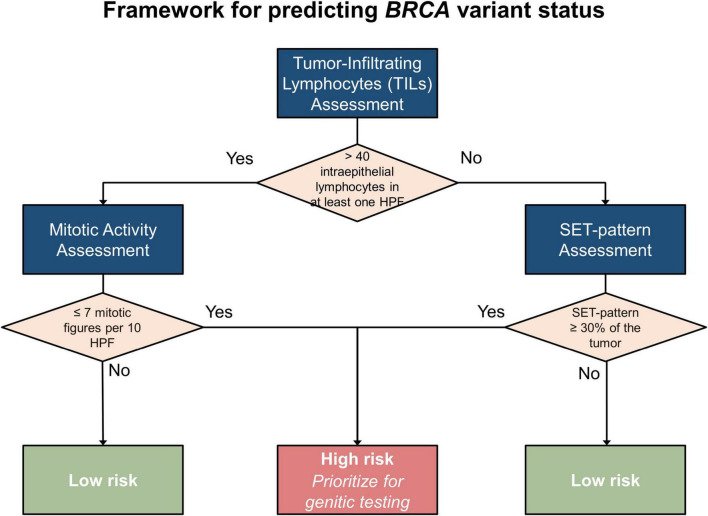
Framework for predicting *BRCA* variant status.

To guide the stratification of cases and prioritize individuals for genetic testing, particularly in resource-limited settings, we developed a framework for predicting the likelihood of pathogenic *BRCA* variants in individual cases of HGSOC based on our findings. The proposed framework (Figure 6) synthesizes the immune context, classified according to TILs, alongside essential pathological characteristics such as SET patterns, necrosis, and mitotic activity.

#### 4.3.1 Step 1: evaluate TILs

To identify hot spots for TILs, the entire tumor should be examined at low-power magnification. Subsequently, the identified areas should be evaluated at high-power magnification. When a single HPF with more than 40 intraepithelial TILs is noted, the tumor should be categorized as TIL-positive. Otherwise, it will be classified as TIL-negative. This stratification is crucial because the diagnostic relevance of specific pathological features varies between TIL-positive and TIL-negative tumors.

#### 4.3.2 Step 2: analyze pathological features by TIL subgroups

Once the immune context is established, specific pathological features can be assessed according to the tumor’s TIL classification:

*In TIL-positive tumors*:

- *Primary marker*: mitotic activity is the most reliable indicator of pathogenic *BRCA* variants in TIL-positive cases.
+ *Low mitotic activity* (≤ 7 mitotic figures/10 HPFs with a 0.5-mm field diameter) is strongly associated with pathogenic *BRCA* variants.
+ *Moderate or high mitotic activity* (> 7 mitotic figures/10 HPFs with a 0.5-mm field diameter) suggests a lower likelihood of pathogenic *BRCA* variants.
- *Secondary features*: although less predictive, SET patterns in ≥ 30% of the tumor or necrosis may provide supportive evidence but should not be relied upon as primary markers in this subgroup. These features are supportive markers because previous studies have identified their correlation with pathogenic *BRCA* variants (29, 30, 36).

*In TIL-negative tumors*:

- *Primary marker*: SET patterns in ≥ 30% of the tumor strongly predict pathogenic *BRCA* variants in TIL-negative tumors.
- *Secondary features*: necrosis and mitotic activity provide Supplementary Information as previous studies have shown their correlation with pathogenic *BRCA* variants (29, 30, 36).

#### 4.3.3 Step 3: synthesize findings

The integration of immune context and pathological features enables stratifying cases into the following two categories:

- *High likelihood of pathogenic BRCA variant*
+ *TIL-positive*: cases with low mitotic activity and supportive features (e.g., necrosis or SET patterns)
+ *TIL-negative*: SET-positive cases, particularly when combined with other supportive markers
- *Low likelihood of pathogenic BRCA variant*
+ *TIL-positive*: cases with moderate or high mitotic activity
+ *TIL-negative*: SET-negative cases

#### 4.3.4 Step 4: prioritize cases for genetic testing

This framework enables prioritizing cases for genetic testing:

- *High-risk cases*: these should be prioritized for NGS or other genetic testing modalities to confirm *BRCA* variant status.
- *Low-risk cases*: testing should be considered only when additional risk factors, including family history and clinical presentation, are present or as resources allow.

### 4.4 Applications of the framework

PARP inhibitors have transformed ovarian cancer treatment, with the SOLO-1, PRIMA, ATHENA-MONO, and PAOLA-1 trials demonstrating significant survival benefits, particularly in patients with *BRCA* mutation and HRD positivity (13). To optimize treatment, early biomarker testing is essential; however, access remains a challenge. By applying our framework, pathologists and oncologists could refine the selection of patients for genetic testing. In particular, histopathological evaluation of TILs and other morphological features may be a cost-effective triage tool in resource-limited settings where universal genetic testing is scarce. Identifying high-risk cases enables efficient use of limited resources, focusing on those most likely to benefit from *BRCA1/2* testing and subsequent interventions. Ultimately, integrating our framework into routine pathological assessments could enhance diagnostic accuracy and guide tailored therapeutic approaches.

### 4.5 Comparison of our proposed framework with other methods for predicting BRCA variant status

Several methods for predicting *BRCA* variant status in HGSOC have been explored, including BRCA1/2 immunostaining and the use of characteristic histopathological features, such as SET patterns, high mitotic activity, necrosis, and TILs (29, 30, 36–38). BRCA1/2 immunohistochemistry, which has the capacity to screen for protein expression loss due to mutation (38), requires specialized reagents and infrastructure. Although some studies have reported a correlation between histopathological features and *BRCA* variant status, the results have been inconsistent, and the association between these features has not been clearly established (18, 28–30).

In contrast, our method solely relies on routine H&E-stained slides, making it more cost-effective than approaches requiring additional immunohistochemical staining. Furthermore, by using a structured framework that combines TIL assessment with other morphological features, such as SET patterns and mitotic activity, our approach more effectively captures the association among tumor morphology, immune microenvironment, and *BRCA* variant status than previous studies.

### 4.6 Limitations and future directions

Our study had several limitations that warrant caution when interpreting the results. First, the sample size was relatively small, especially within the TIL-positive and TIL-negative subgroups, which may restrict the statistical power and generalizability. In addition, this was a retrospective single-center study, potentially introducing selection bias and limiting the applicability of our conclusions to other clinical settings. To validate and ensure that our findings are generalizable to various patient populations, further multi-institutional or prospective studies with standardized protocols for tissue sampling and data collection are warranted. Such a study would help confirm the reliability of the framework and more effectively address confounding factors.

Second, we relied on semiquantitative methods to assess certain morphological features. Although the methods were based on previously published criteria, the lack of a universally accepted and standardized scoring system may result in interobserver variability, thereby affecting reproducibility. Implementing more objective measurement tools, including digital image analysis or standardized scoring guidelines, could improve consistency across different centers and pathologists.

Third, while our study emphasized on associations between pathogenic *BRCA* variants and morphological features, it did not investigate the potential influence of other genetic alterations commonly involved in HGSOC. Genes such as *TP53* (39), which is almost universally mutated in HGSOC, as well as *PTEN* (40), *CCNE1* (41), *BRAF* (42), and other homologous recombination repair-related genes may also shape tumor morphology and promote immune infiltration. The unavailability of molecular data beyond *BRCA1/2* in our cohort represents a limitation in understanding the broader genomic context. Future studies incorporating comprehensive genomic profiling would be valuable in determining how these genetic alterations contribute to specific tumor morphology within immune infiltration.

Finally, although our study emphasized on associations among pathogenic *BRCA* variants, TILs, and mitotic activity, we could not identify a clear relationship among these factors in the existing literature, highlighting a gap in current knowledge. This disparity makes the full contextualization of our results challenging. Further research is warranted to explore the molecular and pathological mechanisms linking immune infiltration, morphological changes, and *BRCA* genetic alterations.

By acknowledging the limitations of our study, we hope to provide context for our findings and encourage future studies that address the noted gaps in methodologies, study design, and mechanistic understanding.

## 5 Conclusion

This study underscores the significance of stratifying HGSOCs by dense or sparse infiltrates of TILs to evaluate the relationship between pathological features and pathogenic *BRCA* variant status. In particular, SET patterns and mitotic activity emerged as critical markers for distinguishing pathogenic *BRCA* variants in TIL-negative tumors and TIL-positive tumors, respectively. These results demonstrate that histopathological evaluation offers a practical and cost-effective method for identifying high-risk patients for molecular analysis, particularly in resource-limited settings. Our method ensures that patients at the highest risk of harboring pathogenic *BRCA* variants receive timely genetic testing and benefit from tailored interventions, including PARP inhibitors, leading to enhanced treatment outcomes.

## Data Availability

The original contributions presented in this study are included in this article/Supplementary material, further inquiries can be directed to the corresponding authors.
