# Supplementary material for: Tumor-infiltrating lymphocytes are the key determinants of pathological features associated with pathogenic BRCA variants in high-grade serous ovarian carcinoma
Source: Front Med (Lausanne). 2025 Jun 3;12:1555883. doi: 10.3389/fmed.2025.1555883 (PMC12172505; doi:10.3389/fmed.2025.1555883)
Supplement: Supplementary file 1 [file Supplementary_file_1.docx]

Supplementary Material

## 1 Supplementary Figure

**
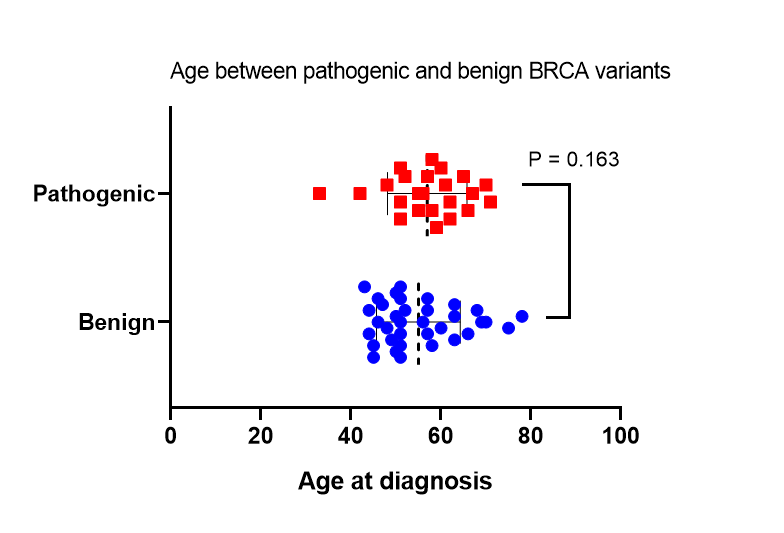
**

**Supplementary Figure 1.** No significant age differences were seen between patients with pathogenic and benign *BRCA* variants.

## 2 Supplementary Table

| Supplementary Table 1. Baseline characteristics of HGSOC patients with and without positive TILs. | | | | |
| --- | --- | --- | --- | --- |
| Characteristic, n(%) | Total  (n = 58) | TIL-positive  (n = 28) | TIL-negative  (n = 30) | *P*-value |
| Age (years) |  | | | |
| <50 | 13(22.41) | 7(25.00) | 6(20.00) | 0.757 |
| ≥50 | 45(77.59) | 21(75.00) | 24(80.00) |  |
| *BRCA* variants |  | | | |
| Benign | 35(34.12) | 19(67.86) | 16(53.33) | 0.294 |
| Pathogenic | 23(65.88) | 9(32.14) | 14(46.67) |  |
| Ovarian laterality |  |  |  |  |
| Unilateral | 36(60.34) | 14(50.00) | 13(43.33) | 0.793 |
| Bilateral | 49(39.66) | 14(50.00) | 17(56.67) |  |
| FIGO stage at diagnosis |  |  |  |  |
| I + II | 2(3.45) | 0(0.00) | 2(6.67) | 0.492 |
| III + IV | 56(96.55) | 28(100.00) | 28(93.33) |  |
| Hemoglobin (missing data = 21) |  |  |  |  |
| Normal | 29(78.38) | 12(75.00) | 17(80.95) | 0.705 |
| Abnormal | 8(21.62) | 4(25.00) | 4(19.05) |  |
| CA125 (missing data = 17) |  |  |  |  |
| <100 | 5(12.20) | 2(11.11) | 3(13.04) | 1.000 |
| ≥100 | 36(87.80) | 16(88.89) | 20(86.96) |  |
| Creatinine (missing data = 12) |  |  |  |  |
| Normal | 37(80.43) | 17(85.00) | 20(76.92) | 0.711 |
| Abnormal | 9(19.57) | 3(15.00) | 6(23.08) |  |
| Albumin (missing data = 20) |  |  |  |  |
| Normal | 34(89.47) | 18(100.00) | 16(80.00) | 0.107 |
| Abnormal | 4(10.53) | 0(0.00) | 4(20.00) |  |
| 1. Categorical variables are presented as numbers(%).  2. *P*-values (two-tailed) were calculated using Fisher’s exact test. *P* < 0.05 is considered to indicate statistical significance.  3. HGSOC: High-grade serous ovarian carcinoma; TILs: tumor-infiltrating lymphocytes. | | | | |
